# Dietary Fat Feeding Alters Lipid Peroxidation in Surfactant-like Particles Secreted by Rat Small Intestine

**DOI:** 10.4021/gr2009.03.1280

**Published:** 2009-03-20

**Authors:** Aasma Turan, Akhtar Mahmood, David H. Alpers

**Affiliations:** a Department of Biochemistry, Panjab University, Chandigarh, 160014, India; b Department of Medicine, Washington University School of Medicine, St. Louis, MO, USA

**Keywords:** Alkaline phosphatase, Fe^2+^/ascorbate system, Microvillus membranes, Dietary oils, Polyunsaturated fatty acids

## Abstract

**Background:**

Long-term feeding of fish oil (n-3) and corn oil (n-6) markedly enhances levels of lipid peroxidation within isolated rat enterocytes. The effect is 10-fold greater at the villus tip than in the crypt region, correlating with the distribution of deleterious oxidative systems (glutathione reductase) in the tip and beneficial systems (superoxide dismutase) at the base of the villus. Because of this vertical gradient of peroxidation, the process was thought to play a role in apoptosis of enterocytes at the villus tip. Surfactant-like particles (SLPs) are membranes secreted by the enterocyte and a component of these membranes is directed to the intestinal surface overlying villus tips. One suggested role for SLPs has been to protect the mucosal surface from the harsh luminal conditions that might enhance apoptotic loss of enterocytes. The hypothesis to be tested was whether SLP lipids, like those in enterocytes, were also peroxidized, although they were external to the cellular processes that seem to oxidize enterocyte lipids, or whether SLP were immune to these biological processes. Feeding with groundnut oil (n-9) was compared with fish oil (n-3) and corn oil (predominantly n-6) to determine whether oils with various lipid composition would affect peroxidation in both SLP and enterocytes.

**Methods:**

After an overnight fast, Wistar rats were fed 2 mL of dietary oil by gavage. Five hours later SLPs and underlying microvillus membranes (MVM) were isolated and analyzed for generation of thiobarbituric acid reactive substances (TBARS) and for hydrolase activities, at baseline and after addition of an Fe^+2^/ascorbate system to induce peroxidation.

**Results:**

In vitro lipid peroxidation using the Fe^2+^/ascorbate system produced greater peroxidation than in MVM. Intestinal alkaline phosphatase (IAP), sucrase and lactase activities were decreased in SLPs, but were unaltered in MVM except for IAP. The activities of maltase, trehalase, Leucine aminopeptidase and γ–glutamyltranspeptidase, were unaffected both in SLPs and MVM under these conditions.

**Conclusions:**

SLPs are more susceptible to oxidative damage than are the underlying MVMs. This may reflect results of a hostile luminal environment. It is not clear whether SLPs are acting as a lipid ‘sink’ to protect the MVM from greater oxidation, or are providing an initial stimulus for apoptosis of villus tip enterocytes, or both.

## Introduction

Free radical species derived from molecular oxygen react with many biological molecules, including all classes of macronutrients [1]. The potential role of lipid peroxidation in the pathogenesis of many disease processes has been supported by a large amount of experimental evidence. Diseases of every organ have been suggested as target of lipid peroxidation, including the intestine, with inflammatory bowel disease being the leading candidate [2]. Lipid peroxidation has been demonstrated to occur at all levels of the intestinal tissue. Salmonella organisms or toxin increased reactive oxygen species (ROS) in mucosal macrophages as well as in enterocytes [3]. *Escherichia coli O157I* derived verotoxins produce increased ROS from mucosal endothelium, a finding that has been suggested to be a factor in the vasculitis (hemolytic-uremic syndrome) that can occur with infection [4]. The enterocytes themselves, and even the apical membranes of enterocytes and colonocytes, can be altered ex vivo by superoxides [5]. Moreover, alkaline phosphatase activity is very susceptible to damage by peroxidation, whereas other microvillus membranes (MVM) hydrolases are not [5, 6].

Surfactant-like-particle (SLP) is a specialized membrane that is secreted from enterocytes, colonocytes and gastric epithelial cells in mammalian intestine [7, 8]. The membranes are rich in alkaline phosphatase and exhibit surface tension lowering activity [9, 10]. The majority of earlier studies have been carried out in animals fed corn oil for 3 - 5 hours, a time at which the SLPs are maximally produced and migrate to the surface of the epithelial cells [9]. SLPs also contain proteins (SP-A, SP-D) found in pulmonary surfactants.

The enterocytes along the vertical axis of the intestinal villus demonstrate a gradient of lipid peroxidation, with the tip cells after 30 days of fat feeding with three different dietary oils showing markedly more peroxidation induced ex vivo by Fe^2+^/ascorbate and as measured by malondialdehyde [11, 12]. Moreover, the free radical scavenger enzymes, catalase and superoxide dismutase showed the highest activity in the crypt cells compared to cells at the villus tip. In contrast, the prooxidant enzymes glutathione transferase and glutathione reductase were 2 - 5 times higher in the villus tip. These studies assessed the status of the enterocyte, but not that of the SLPs overlying the enterocyte. The feeding of different dietary fats is known to induce changes in the chemical composition of cell membranes in various tissues [13, 14], possibly altering the level of lipid peroxidation. Kumar et al [15] reported alterations in the chemical composition of SLPs after ingestion of different dietary oils, and in addition noted that both fish oil and olive oil (n-9) stimulated the in vivo secretion of SLPs, for olive oil even more than corn oil. Therefore, the current study was performed to answer two questions: First, to see whether acute fat feeding of representative dietary oils (n-3, n-6, and n-9 predominant) could reproduce the effects on lipid peroxidation of chronic fat feeding; second, to determine whether the extracellular membrane, SLPs, also demonstrate these changes.

## Materials and Methods

### Experimental design

Male Albino rats (Wistar strain) weighing 90 - 100g obtained from the central animal house of Panjab University, Chandigarh were kept on standard rat pellet diet (Hindustan Lever Ltd., Ghaziabad, India). Animals were randomized into four dietary groups according to the dietary fat fed with Fish oil, Corn oil, Groundnut oil or saline. A day before sacrifice, animals were fasted overnight, giving free access to water. The fasted animals were given either 2 ml of the respective oil or normal saline by Ryles tube without anesthesia. Animals were sacrificed 5 hours after fat feeding under light ether anesthesia. The experimental protocol was approved by the Ethical Committee of the Institute on the use of laboratory animals. Experiments on animals were performed in accordance with guidelines for use of laboratory animals, approved by Indian Council of Medical Research, New Delhi.

### Isolation of SLPs

SLPs were isolated by the method of Eliakim et al [9] and purified using a Sepharose 6B column and 10 mM Tris-HCl buffer, pH 7.4 [16]. Light Mucosal scrapings collected from intestinal lumen by Whatman paper No. 3 were suspended in Tris-HCl buffer (5 mM, pH 7.4), containing 5 mM CaCl_2_ and 150 mM NaCl. The suspension was sonicated for 20 seconds at optimum speed or vortexed briefly, followed by centrifugation at 600g for 10 min to remove the remnants of filter paper. One ml of the resulting supernatant fraction was loaded onto a Sepharose 6B column (1.5 x 30 cm) and 2 ml fractions were collected. Alkaline phosphatase, a marker of SLPs, was assayed in each fraction. Fractions 9 - 12 containing SLP eluted prior to the void volume, were pooled and used for various biochemical studies. All procedures were done at 4°C, except otherwise stated.

### Preparation of MVMs

The intestinal mucosa was removed from the muscle layer, by scraping with glass slides. MVMs were isolated by calcium chloride precipitation method as described by Kessler et al [17]. The purity of the membranes was determined by assaying the activity of the marker enzyme, sucrase. The final membrane preparation exhibited 12 to 15-fold enrichment of brush border sucrase over the crude homogenate. The membrane preparation was used as such, for various biochemical studies

### Assay of brush border enzymes

Alkaline phosphatase (AP) activity was determined by the method of Bergmeyer [18]. Sucrase, lactase, maltase and trehalase activities were assayed by measuring D-glucose liberated from the respective disaccharides, using a glucose-oxidase-peroxidase method modified from the procedure described by Dahlqvist [19]. The activity of leucine aminopeptidase (LAP) was measured according to Goldberg and Ruttenberg [20], using L-leucine-p-nitroanilide as the substrate. The activity of γ- glutamyl transpeptidase (γ-GTP) was assayed by the method of Naftalin et al [21] and γ-glutamyl p-nitroanilide hydrochloride was used as the substrate. Protein was estimated by the method of Lowry et al [22] using bovine serum albumin as the reference standard. All the enzyme activities were expressed as units per milligram of protein. One enzyme unit is defined as the amount of enzyme, which can transforme 1 mmole of substrate to product per minute under the standard assay conditions.

Separation of SLP membrane proteins solubilized with 0.1% sodium dodecyl sulfate (SDS) was carried out by polyacrylamide gel electrophoresis (PAGE) under non-denaturing conditions [23]. One hundred micrograms of the SDS solubilized membrane protein was run on 8% acrylamide gels. AP activity was stained in situ in presence of 3’-bromo-4-chloro indolyl phospharte (BCIP, 1 mg/ml in 50 mM Tris-HCl, pH 7.6) solution at 37 °C. After bands of desired color intensity were obtained, the reaction was stopped by transferring the gels to 10% acetic acid. Finally the gel was washed with distilled water and dried under vacuum in gel dryer at 80°C for 2 - 3 h using cellophane membranes.

### Induction of lipid peroxidation in membranes

Membranes (approximate 1 mg membrane protein) were aerobically incubated in 1 ml of 10 mM Tris-HCl buffer, pH 7.4, in a shaking water bath at 37°C for 60 minutes in the presence or absence of Fe^2+^/ascorbate system as described by Fodor and Marx [24]. For experiments designed to assess the effects of lipid peroxidation on enzyme activities in MVM, the reaction was terminated by dilution with 40 ml of 10 mM Tris-HCl buffer, pH 7.4, at 0°C, followed by centrifugation at 43,000g for 30 minutes. The pellets were washed twice with the same buffer. SLP were dialyzed for 6 h in 20 mM Tri-HCl buffer pH 7.4, at 4°C, twice. For experiments designed to measure thiobarbituric acid (TBA), the reaction was terminated by 3(2)-t-butyl-4-hydroxyanisole (BHA, 5 mM) made in an ethanolic stock. Thiobarbituric acid reactive substance (TBARS) were measured in SLPs and MVM, incubated with the Fe^2+^/ascorbate system using the TBA assay as described by Ohkawa et al [25].

### Analysis of lipids from surfactant-like particles

Extraction of lipids from surfactant like particles was carried out following the method of Folch et al [26]. Phospholipid phosphorus was estimated by the method of Marinetti [27]. Total cholesterol was estimated colorimetrically using glacial acetic acid-FeCl_3_ reagent as described by Zlatkis et al [28].

### Statistical analysis

All data were expressed as means ± SD, statistical analysis of the data was done using paired students *t* test, p value less than 0.05 was considered statistically significant.

## Results

Feeding fish oil (n-3) to rats, enhanced intestinal alkaline phosphatase (IAP) activity by 35%, corn oil by 64% or groundnut oil by 135% in SLP compared to control group (Table 1). However, MVM obtained from corn oil or groundnut oil fed rats revealed a decline (p < 0.001) in IAP activity, compared to control membranes, whereas feeding fish oil showed 30% increase in enzyme activity under these conditions. Activity of the other hydrolases were present at much lower levels in SLP compared with IAP, and perhaps for this reason, it showed more variability in percent change after fat feeding; sucrase activity was decreased in SLP isolated from fish oil, corn oil, or groundnut oil-fed rats, but only those fed groundnut oil showed a significant (p < 0.01) decrease in enzyme levels. MVM obtained from fish oil, corn oil or groundnut oil fed rats exhibited a significant decrease in sucrase activity in brush borders compared to controls. The activity of lactase was reduced after fat feeding in SLP and MVM, except for MVM from groundnut oil fed animals, where an increase (p < 0.001) in lactase activity was found. The activities of maltase (40 - 75%) and trehalase (59 - 81%) were reduced after fat feeding in SLP and MVM (Table 1). Overall, feeding any of the three dietary oils led to decreases in disaccharidase activity in both SLP and MVM.

**Table 1:** Distribution of brush border enzymes in surfactant-like-particles and microvillus membranes from control and oil fed rats.

Enzyme	SLP				MVM			
Protein (Units/mg)	Control	Corn oil	Fish oil	Groundnut oil	Control	Corn oil	Fish oil	Groundnut oil
AP	2.73 ±0.18	4.48±0.15***	3.69±0.10***	6.38±0.05***	2.170±0.18	1.19±0.09***	2.82±0.05***	1.62±0.13^**^
		(+64%)	(+35%)	(+135%)		(-45%)	(+30%)	(-25%)
Sucrase	0.151±0.03	0.122±0.06^NS^	0.135 ±0.05^NS^	0.083±0.02^**^	2.27±0.12	0.851±0.05***	1.89±0.14^**^	0.78±0.08***
		(-19%)	(-11%)	(-45%)		(-62%)	(-17%)	(-66%)
Lactase	0.011±0.001	0.005±0.001	0.007±0.001^*^	0.004±0.001***	0.074±0.005	0.017±0.001^**^	0.056±0.001***	0.115±0.005***
		(-55%)	(-36%)	(-66%)		(-77%)	(-24%)	(+55%)
Maltase	0.163±0.008	0.045±0.005***	0.072±0.005***	0.097±0.005***	0.756±0.05	0.358±0.06***	0.185±0.04***	0.301±0.07***
		(-72%)	(-56%)	(-40%)		(-53%)	(-75%)	(-60%)
Trehalase	0.128±0.002	0.025±0.001***	0.042±0.003***	0.033±0.001***	0.886±0.03	0.183±0.06***	0.195±0.05***	0.201±0.02***
		(-80%)	(-67%)	(-74%)		(-79%)	(-78%)	(-77%)
LAP	0.120±0.01	0.041±0.01***	0.053±0.01***	0.059±0.005^*^	0.235±0.005	0.251±0.06^NS^	0.17±0.003***	0.185±0.003***
		(-66%)	(-55%)	(-51%)		(+7%)	(-28%)	(-21%)
γ- GTP	0.117±0.005	0.063±0.003***	0.134±0.03^NS^	0.082±0.004***	0.268±0.01	0.237±0.04^NS^	0.301±0.01^N S^	0.291±0.05^NS^
		(-46%)	(+17%)	(-30%)		(-12%)	(+12%)	(+9%)

***p < 0.001; ^**^p < 0.01; ^*^p < 0.05; NS = non significant. Values are mean ± SD, n = 8.

LAP activity was low (p < 0.001) in SLP isolated from fish oil, corn oil, or groundnut oil fed animals compared to control SLP. The γ-GTP activity remained unaffected in SLP isolated from fish oil fed animals, while SLP isolated from corn oil or groundnut oil fed animals exhibited reduced (p < 0.001) γ-GTP levels. LAP activity (p < 0.001) was low in MVM obtained from fish oil or groundnut oil fed rats, but the levels of γ-GTP were unaffected under these conditions (Table 1).

Phospholipid (PL) and cholesterol contents were enhanced in response to feeding different dietary oils, varying in unsaturated fatty acids, i.e. corn oil (ω-6), fish oil (ω-3) or groundnut oil (ω-9). The Chl/PL molar ratio was 0.73 in saline fed rats, 0.62 in corn oil, 0.64 in fish oil or 0.68 in groundnut oil fed animals, which was far below that of 1.49 in microvillus membranes (results not shown). Feeding fish oil, corn oil or groundnut oil elevated serum AP levels by 324%, 297% and 395%, respectively, compared to controls. Serum triglyceride levels (9 mg/dl) were elevated (262.2 ± 11) after feeding fish oil, corn oil (297.2 ± 12) or groundnut oil (246.4 ± 11) to rats compared to control rats. Thus, no pattern of changes in serum lipids were noted that were specific for one of the three dietary fats.

**Figure 1:**
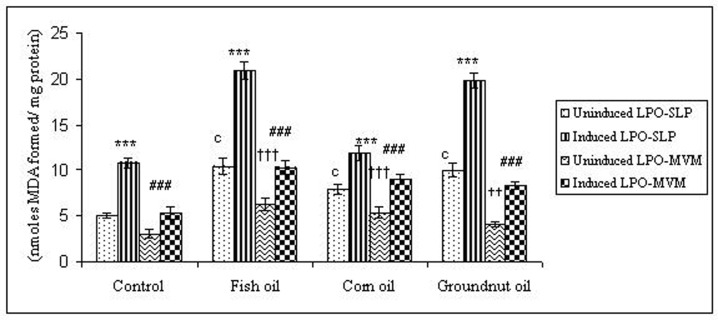
Lipid peroxidation levels induced under *in vitro* (Fe^2+^/ ascorbate system) in response to feeding different dietary fats in SLP and MVM. 300-400µg of membrane protein was used for each assay. Values are mean ± SD, n = 8. Uninduced LPO-SLP vs Induced LPO-SLP, * p < 0.05; **p < 0.01; p < 0.001. Uninduced LPO-MVM vs Induced LPO-MVM, a, p < 0.05; b, p < 0.01; c, p < 0.001. Control SLP vs Different dietary oils SLP MVM, # p < 0.05; ## p < 0.01; ### p < 0.001. Control MVM vs Different dietary oils, † p < 0.05; †† p < 0.01; ††† p < 0.001.

**Figure 2:**
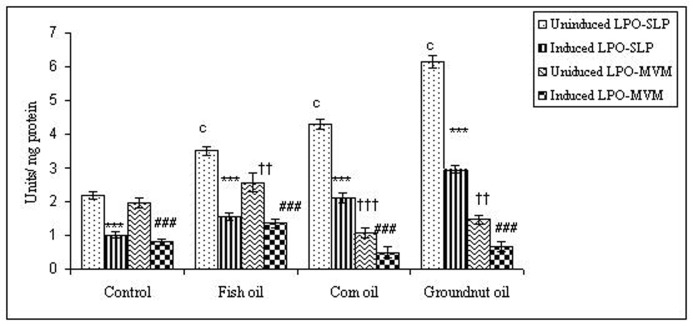
Effect of lipid peroxidation induction *in vitro* on IAP activity (µmole/min/mg protein) of SLP and MVM isolated from control and fat fed rats. Values are mean ± SD, n = 8. Uninduced LPO-SLP vs Induced LPO-SLP, *p < 0.05; **p < 0.01; ***p < 0.001. Uninduced LPO-MVM vs Induced LPO-MVM, a, p < 0.05; b, p < 0.01; c, p < 0.001. Control SLP vs Different dietary oils SLP, #p < 0.05; ##p < 0.01; ###p < 0.001. Control MVM vs Different dietary oils MVM, †p < 0.05; ††p < 0.01; †††p < 0.001.

**Figure 3:**
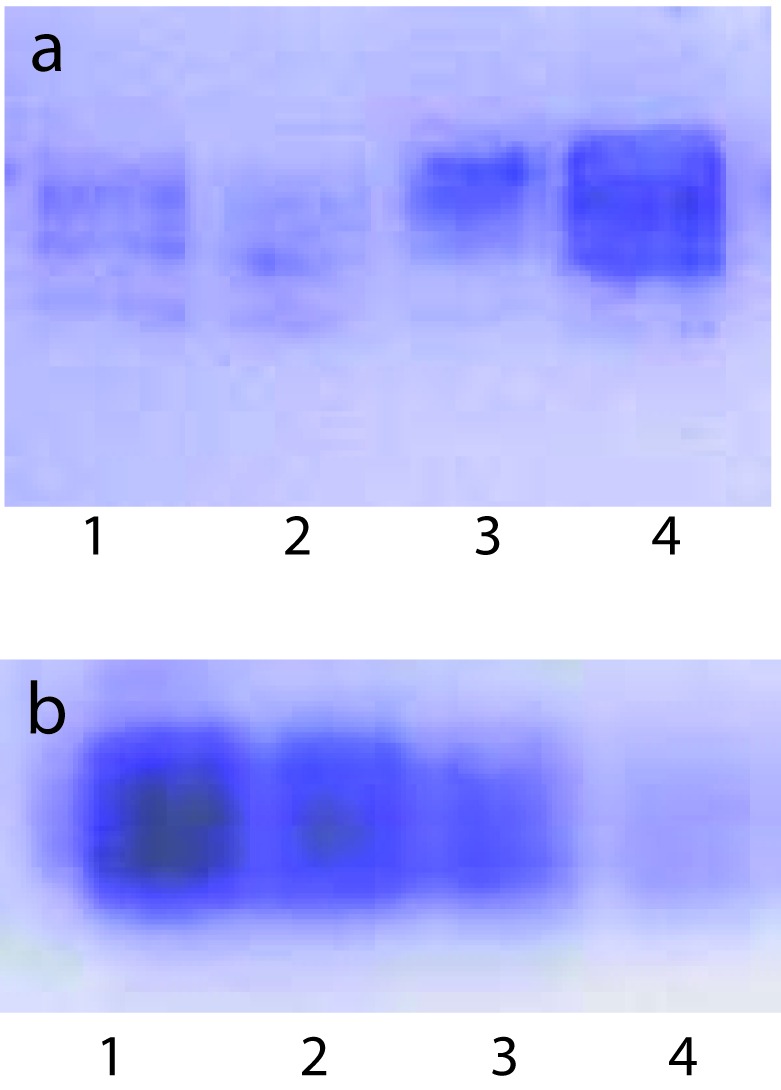
BCIP - staining of AP activity upon lipid peroxidation induced *in vitro* in SLPs isolated from control and oil fed animals under these conditions. Each lane contained 100 µg protein. Each lane contained 100 µg SLP protein. (a), 1, Induced Control; 2, Induced Fish Oil; 3, Uninduced Control; 4, Uninduced Fish Oil. (b), 1, Uninduced Groundnut Oil; 2, Induced Groundnut Oil; 3, Uninduced Corn Oil; 4, Induced Corn Oil.

**Figure 4:**
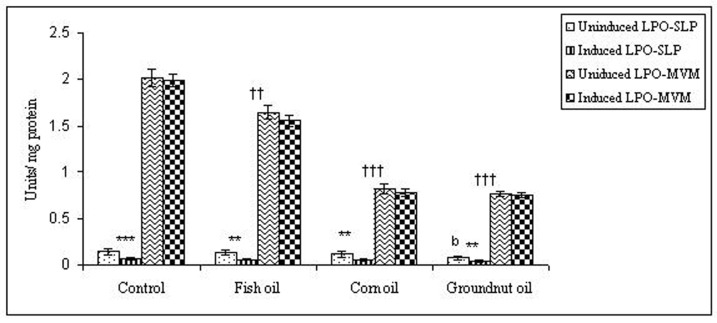
Effect of lipid peroxidation induction *in vitro* on sucrase activity (µmole/min/mg protein) of SLP and MVM isolated from control and fat fed rats. Values are mean ± SD, n = 8. Uninduced LPO-SLP vs Induced LPO-SLP, *p < 0.05; **p < 0.01; ***p < 0.001. Uninduced LPO-MVM vs Induced LPO-MVM, a, p < 0.05; b, p < 0.01; c, p < 0.001. Control SLP vs Different dietary oils SLP, #p < 0.05; ##p < 0.01; ###p < 0.001. Control MVM vs Different dietary oils MVM, †p < 0.05; ††p < 0.01; †††p < 0.001.

**Figure 5:**
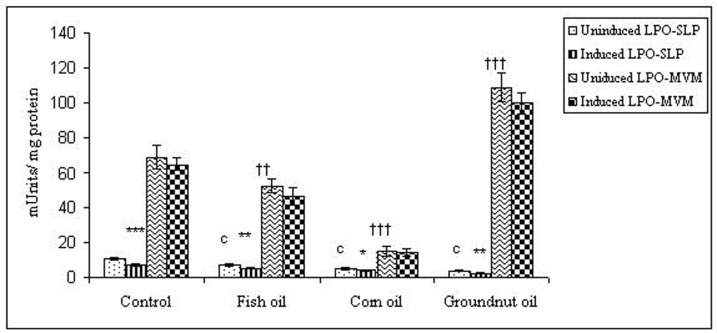
Effect of lipid peroxidation induction *in vitro* on lactase activity (µmole/min/mg protein) of SLP and MVM isolated from control and fat fed rats. Uninduced LPO-SLP vs Induced LPO-SLP, *p < 0.05; **p < 0.01; ***p < 0.001. Uninduced LPO-MVM vs Induced LPO-MVM, a, p < 0.05; b, p < 0.01; c, p < 0.001. Control SLP vs Different dietary oils SLP, #p < 0.05; ##p < 0.01; ###p < 0.001. Control MVM vs Different dietary oils MVM, †p < 0.05; ††p < 0.01; †††p < 0.001.

To examine whether lipid peroxidation of SLP affected associated enzyme levels, the Fe^2+^/ascorbate oxygen radical generating system was used to induce lipid peroxidation under *in vitro* conditions (Fig. 1). The formation of malondialdehyde (MDA) was used as a measure of the rate of lipid peroxidation and an index of membrane damage. As shown in Fig. 2, the activity of IAP was reduced (p>0.001) upon lipid peroxidation both, in SLP and MVM. This was also evident by low intensity of the signal of AP activity upon BCIP-staining in acrylamide gels, under non-denaturing conditions (Fig. 3). The activities of sucrase, lactase, maltase and trehalase were unaffected under *in vitro* lipid peroxidation induced conditions in MVM (data not shown). But, in the SLPs, activities of sucrase and lactase were reduced by 26 - 58%, while those of maltase and trehalase were unaffected (Fig. 4, 5). The activities of LAP and γ-GTP were also unaltered upon lipid peroxidation in both SLP and MVM, in control and oil fed animals (data not shown).

## Discussion

SLPs isolated from groundnut oil fed animals showed an increase in AP activity, but a decrease in microvillus membranes, which is similar to that seen after feeding corn oil to rats [29], while fish oil containing n-3 fatty acids exhibited an increase in AP activity (units/mg protein) in both SLP and MVM compared to control animals. Stenson et al [30] showed that feeding coconut or fish oil to animals altered brush border AP activity, while opposite effects of feeding corn and olive oil have been reported by Brasitus et al [31]. These differences in AP concentration in MVMs cannot be resolved with the available data. The present study confirms the increased AP concentration in SLP with only modest effect on MVM that was documented earlier after feeding rats with corn oil, and also shows that groundnut oil provides an even larger effect. A number of factors which may be responsible for these observed differences in AP concentration in SLP include differential effects on the expression of SLP proteins or on the rate of assembly of the lipoprotein particles, or the luminal stability of the particles. Moreover, the difference in response to enzyme concentrations in the MVM would support the concept that SLP are not degraded products of microvillus membrane but are a distinct entity [32].

Disaccharidases are present in very low concentration in SLP membranes, as reported by Engle et al [33]. A decrease in enzyme levels after feeding any of the three oils was the only consistent pattern seen. Kaur et al [13] have shown that chronic feeding of fat produced changes in lipid composition of microvillus membranes. Since feeding of different dietary oils reduced the enzyme levels in brush borders, an altered lipid composition of the SLP could explain these findings.

A strong connection between lipid droplets and lamellar structures after a fatty meal exists, where SLP appears to surround the intracellular lipid droplets, which is depleted during Pluronic L-81 treatment [29]. Grewal and Mahmood [34] reported that administration of actinomycin D or cycloheximide to fat-fed animals reduced the concentration of IAP in SLP and MVM, and prevented its appearance in the serum in response to fish oil. Thus coordination between lipid and protein synthesis is necessary for the formation of SLP, which acts as a vehicle for the transport of IAP into the circulation [35]. Recently, Kaur et al [36] reported that, irrespective of the type of fat feeding, triacylglycerol modulates IAP and SLP secretions. Young et al [37] also suggested that coordination between protein synthesis and lipid transport is a prerequisite step for the formation of SLP and hence the transport of IAP into the circulation.

Dietary intake of unsaturated fats contributes significantly to lipid peroxides in the intestinal lumen [38]. In animals, toxicity of dietary polyunsaturated oils correlated with their peroxide contents which act on intestinal mucosal cells after transport into those cells [39]. Similar toxic luminal concentrations have not yet been demonstrated in humans. However, the diet contains many pro-oxidants (e.g. iron, copper, heme, lipid and other peroxides and aldehydes) and antioxidants as well [40]. In addition, some reduced compounds (e.g. vitamin K) can be transported into the colonocyte where they can be involved in the oxidation of other intracellular components [41]. Thus, the intestinal mucosa is exposed daily to peroxides and other pro-oxidant compounds that may act in the lumen or after transport into the epithelial cells [42].

Induction of lipid peroxidation in SLP and MVM by the Fe^2+^/ascorbate oxygen-radical generating system reduced activities of sucrase and lactase, while those of maltase and trehalase were unaltered. This difference could be due to different orientation of digestive enzymes in SLP or MVM. The activities of LAP and γ-GTP in both SLP and MVM were unaffected by lipid peroxidation. In contrast to above observations, AP activity was the most impaired (p < 0.001) during lipid peroxidation in vitro, both in SLP and MVM. The method used to detect lipid peroxidation in this study was measurement of MDA by the thiobarbituric acid-reacting substance assay. This test measures MDA generated by decomposition of lipid peroxides during the acid-heating stage of the test, rather than measuring free MDA [1]. It has been widely used for ex vivo studies in animals, as the system is not contaminated with MDA produced in vivo by platelets.

Lipid peroxidation can have some beneficial effects on cells (e.g. eicosanoid synthesis, cell maturation), but also deleterious ones, such as membrane disruption and cellular dysfunction [43]. The balance within cells is determined by peroxidizing and peroxide reducing enzymes. Within the intestinal mucosa not all the enzymes that generate and destroy peroxides are well defined. One of the well characterized enzymes in the rat intestine is the major glutathione peroxidase, a selenium-dependent enzyme that is gut specific (GSHPx-GI) [44]. Its activity is maximal in the ileum and colon. This activity is evenly distributed along the vertical crypt-villus axis of the small intestine in the rat [45]. However, other antioxidant enzymes, such as catalase and superoxide dismutase, are more abundant in the crypt region [11, 12]. Thus, the tips of the villi may be more susceptible to lipid peroxidation than are the crypt cells. The role played by the SLP in this system is not yet clear. Antioxidant systems have not been identified in SLP, and dietary substances with antioxidant activity do appear in the lumen (e.g. carotenoids, tocopherols), but it is not clear if their luminal concentration is sufficient for biological efficacy. The SLP may, therefore, be more exposed to luminal peroxides and would be damaged as in the in vitro Fe/ascorbate system used in the present study. On the other hand, SLP may act as a free radical sink to detoxify peroxides in the lumen or generated by villus tip enterocytes that have a relatively impaired antioxidant capacity. Either way, SLP appears to be caught in between the hostile luminal environment and the villus tip enterocytes at risk for apoptosis. Further studies will be needed to dissect the possible roles of SLP in the peroxidation process. Although unsaturated fat feeding may increase the risk for peroxidation of both SLP and MVM, the type of unsaturated fat does not seem to be a major factor in regulating this complex system.

In conclusion, SLPs are more susceptible to oxidative damage than are the underlying MVMs. This may reflect results of a hostile luminal environment. It is not clear whether SLPs are acting as a lipid ‘sink’ to protect the MVM from greater oxidation, or are providing an initial stimulus for apoptosis of villus tip enterocytes, or both.
